# The effects of add-on self-care education on quality of life and fatigue in gastrointestinal cancer patients undergoing chemotherapy

**DOI:** 10.1186/s12906-019-2800-5

**Published:** 2020-01-16

**Authors:** Jun Xie, Tingli Zhu, Qun Lu, Xiaomin Xu, Yinghua Cai, Zhenghong Xu

**Affiliations:** 1 Department of Oncology, Wuxi People’s Hospital Affiliated to Nanjing Medical University, Wuxi, 214023 China; 2 Nursing Department, Wuxi Children’s Hospital, Wuxi, 214023 China; 3 Internal Medicine Department, Wuxi People’s Hospital Affiliated to Nanjing Medical University, Wuxi, 214023 China

**Keywords:** Gastrointestinal cancer, Self-care, Chemotherapy, Quality of life, Fatigue

## Abstract

**Background:**

Gastrointestinal cancer is one of the most common malignancies and imposes heavy burdens on both individual health and social economy. We sought to survey the effect of a self-care education program on quality of life and fatigue in gastrointestinal cancer patients who received chemotherapy.

**Methods:**

Ninety-one eligible gastrointestinal cancer patients were enrolled in this study and 86 valid samples were analyzed. Data were acquired with a demographics questionnaire, endpoint multidimensional questionnaire and the European Organization for Research and Treatment of Cancer (EORTC) quality of life questionnaire QLQ-C30. The collected data were analyzed using SPSS software.

**Results:**

The self-care education intervention significantly improved the quality of life with respect to emotional function (*p* = 0.018), role function (*p* = 0.041), cognitive function (*p* = 0.038) and alleviated side effects such as nausea/vomiting (*p* = 0.028) and fatigue (*p* = 0.029). Further analysis demonstrated that the self-care education benefited total fatigue, affective fatigue and cognitive fatigue in gastrointestinal cancer patients regardless of baseline depression.

**Conclusion:**

Our results suggested the beneficial effects of the self-care education in both quality of life and anti-fatigue in gastrointestinal cancer patients under chemotherapy. The self-care education could be considered as a complementary approach during combination chemotherapy in gastrointestinal cancer patients.

## Background

Gastrointestinal cancer consists of malignant conditions in the gastrointestinal tract and accessory organs of digestion, including the esophagus, stomach, biliary system, pancreas, small intestine, large intestine, rectum, and anus, which represent the most common malignancies worldwide with persistently increasing mortality and morbidity [1]. According to the cancer statistics, gastrointestinal cancer accounts for 38% of all human cancers and about 45% of mortality rate [2]. The etiology of tumors originated in the gastrointestinal system strongly associates with lifestyle, environment and genetic abnormalities [3]. The quality of life is badly compromised with disease progression and the overall prognosis is relatively unfavorable [4]. Despite increased understanding of the cancer biology underlying this malignancy and extensive exploitation for clinical treatment, chemotherapies are still the mainstay approaches with relative effectiveness [5]. However, most patients suffer severe side effects intrinsically linked to chemotherapy drugs and compromised general well-being, such as weakness, dizziness, lack of appetite, nausea, vomiting, anemia and nutrition disorder [6]. Notably, the elicited psychological stress tremendously deteriorates disease progression as well, which jointly leads to decreased quality of life in cancer patients. The severity of side effects of chemotherapy and affected general well-being varies from individual to individual, and in some cases, severe side effects impede patients from further medical treatment [7]. Social behaviors are also heavily impaired by the severe side effects, which frequently manifest as disrupted social roles, social isolation and depression. Therefore, in addition to the standard therapies, remedies to control chemotherapy-associated side effects are critical for clinical care of cancer patients to improve general well-being.

General medicines have been exploited for this purpose, and serotonin, corticosteroids and metoclopramide receiver antagonists are usually prescribed to treat nausea and emesis after chemotherapy [8]. However, about half receivers show no response to these medicines [9]. Furthermore, these medicines are expensive and present their own side effects including blood pressure decline, headache, constipation, fatigue and diarrhea, which highlight the importance of complementary medicines in both improving quality of life for these patients and benefiting the health system. A variety of non-pharmaceutical methods are proposed and experimented for this purpose, including traditional medicine, muscle relaxation, hypnosis and acupuncture [10]. Among them, the self-care theory proposed by Dorothea Orem highlights the availability and accessibility of self-care to reduce side effects of chemotherapy outside any medical institution [11], which has been introduced since 1959 to describe the role of the nurse in helping a person experiencing inabilities in self-care. The goal of the Orem system is to meet the patient’s self-care demands until the family and/or patient is capable of providing care. The beneficial effects of the self-care education are extensively practiced worldwide, however, few studies have been performed to address the systematic impact on the quality of life and control of side effects in gastrointestinal cancer patients. Here we sort to evaluate the overall effect of a self-care education on control of side effects in gastrointestinal cancer patients under chemotherapy.

## Methods

### Participants

**Fig. 1:**
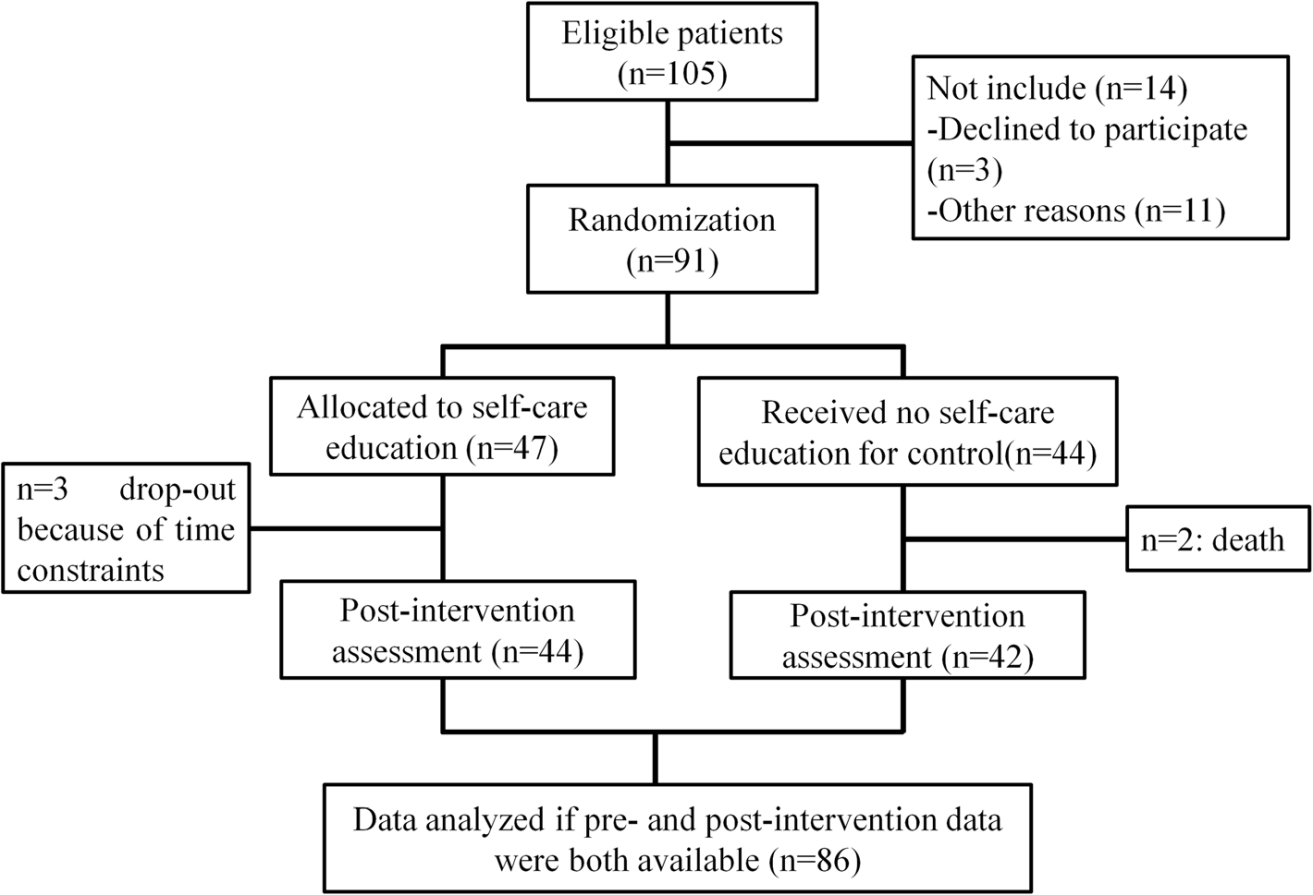
Research framework of this study

The randomized clinical trial was conducted in the chemotherapy department of Wuxi People’s Hospital Affiliated to Nanjing Medical University between June 2016 to October 2017. Official approval was obtained from the Ethics Committee of Wuxi People’s Hospital Affiliated to Nanjing Medical University. The gastrointestinal cancer patients who met the inclusion criteria and signed the informed consent were enrolled in this study. The inclusion criteria employed here were adults with a definite diagnosis of gastrointestinal cancer, and receiving either single or combination chemotherapy, without previous self-care education, without digestive disease/kidney disease/liver failure/gastrointestinal tract obstruction. The exclusion criteria included: 1) reluctance to participate in the study; 2) participation in other educational courses held in the center; 3) cognitive limitations to understand the study protocol and answer the questions asked. The primary endpoint of this study was changes in the quality of life between pre- and post-intervention. In consideration of the possible drop-out, the patient enrollment was terminated after 91 cases were randomly assigned into both groups. The patients who met the inclusion criteria while declined participation were excluded from the sample group. The subjects were randomly allocated into the intervention and control groups (Fig. 1). In our study, all the patients received continuous chemotherapy until the end of the intervention. In total, 105 patients fulfilled the inclusion criteria and 101 were invited to participate but 3 of them declined to participate. Researchers were unable to contact 7 patients before their first chemotherapy. Therefore, 91 agreed to participate and started the follow-up period (47 in the self-care education group and 44 in the control group). We administered the questionnaires at the same time before and after the intervention. After a 12-week intervention (see Additional file 1), there were 3 and 2 missed in the two groups, respectively, so 44 remained in the self-care education group and 42 in the control group. We analyzed the data if pre- and post-intervention data were both available.

### Intervention

The education stage was performed in the oncology ward of the hospital by the well-trained nurses in strict accordance with the pre-organized courses to ensure consistency. The enrolled patients were briefly informed about the global design of self-education. A package of self-care measures including muscular progressive relaxation, music, and education on nutrition was adopted from a previous study [12]. The demographics investigation was performed firstly and initial quality of life was scored with respect to the severity of nausea and emesis. Afterward, the education section composed of a 12-session (45–60 min) course employing slides and videos, which emphasized three major aspects including muscular progressive relaxation, distraction and nutrition optimization referred to the previous reports [12] implicating the beneficial effects on improvement of patient’s physical and mental status. To reduce the severity of nausea and emesis, a muscular progressive relaxation technique was trained to the patients in the experiment group as a self-care measure. Another self-care measure was distracting the mind by listening to music before, during, and after the chemotherapy. We instructed the finger massage, integrated music appreciation and meditation classes regularly. One of the main elements of the self-care education was training about nutrition. The diet adjustment suggestion was offered as a dry, carbohydrate-rich, protein-rich, vitamin-rich recipe. The training was performed two months immediately before chemotherapy.

### Data collection and measures

Quality of life questionnaire was performed with the modified European Organization for Research and Treatment of Cancer (EORTC) quality of life questionnaire QLQ-C30 version 3.0 for all randomized Patients, which comprised 5 functioning scales (Physical, Role, Emotional, Cognitive, and Social), with higher score indicating better quality of life, and 3 symptom scales (Fatigue, Nausea/Vomiting, and Pain) and 2 single-item scales (Appetite Loss and Diarrhea), with higher score indicating poorer quality of life. Fatigue was self-assessed by the patients using the validated 20-item multidimensional Fatigue Assessment Questionnaire covering the dimensions of physical, affective and cognitive fatigue. The fatigue degree was scored by integration the appropriate items (0 = none, 1 = mild, 2 = moderate and 3 = strong), which high value indicating worse fatigue. Depression was self-evaluated following the guide of 20-item Center for Epidemiologic Studies Depression scale (CES-D). The higher scores indicated more severe symptoms. The questionnaire was collected before and after self-education, respectively.

### Data analysis

After collecting the questionnaires, all data were processed and analyzed using the SPSS 23.0 software. The sample size was determined using established statistical power analysis [13]. Differences between means of each compared treatment groups were divided by the standard deviation to determine the standardized effect size, then using 5% as significance level in the student *t* test and 90% power, the minimum required sample size was calculated as previously described [12], which was sufficient for our current sample size after consideration of dropout. The difference between every parameter was analyzed using the student *t* test or *chi* square test. The *p* value was calculated and *p* < 0.05 was considered as significantly different.

## Results

As shown in Table 1, the average age of the participants in the control and self-care education groups were 53.4 ± 9.7 and 52.9 ± 10.4 years, respectively. The male patients accounted for 52.27% in the self-care education group, versus 50% in the control group. Results of independent *t*-test (*p* = 0.61, *p* = 0.52) showed no significant differences between the two groups in terms of both age and gender. Moreover, the demographic characteristics displayed no significant differences between the participants with respect to body weight, body mass index (BMI), baseline depression, smoking history/status, tumor sites, tumor stage, surgical resection and chemotherapy history/regimen.

**Table 1:** Demographic and clinical characteristics of the patients analyzed

Characteristics	Study group		*p*
	Self-care education (*n* = 44)	Control (*n* = 42)	
Age (years)	53.4 ± 9.7	52.9 ± 10.4	0.61
Male gender	23 (52.27%)	21 (50%)	0.52
Body weight (kg)	70.6 ± 11.3	71.1 ± 12.4	0.86
BMI (kg/m^2^)	25.9 ± 4.2	26.2 ± 4.6	0.64
Baseline depression score	24.6 ± 13.3	16.1 ± 11.6	0.31
Baseline depression			0.45
No (score ≤ 38)	33 (75%)	34 (80.95%)	
Yes (score ≥ 38)	11 (25%)	8 (19.05%)	
Smoking year before diagnosis	13 (29.55%)	8 (19.05%)	0.21
Still smoking at baseline	4 (9.09%)	5 (11.9%)	0.84
Tumour site			0.72
Rectum	10 (22.73%)	12 (28.57%)	
Colon	25 (56.82%)	21 (50%)	
Gastric	9 (20.45%)	9 (21.43%)	
Tumour Stage TNM			0.61
II	7 (15.91%)	9 (21.43%)	
III	23 (52.27%)	20 (47.62%)	
IV	14 (31.82%)	13 (30.95%)	
Previous tumour resection	35 (79.55%)	32 (76.19%)	0.48
Time between tumour resection and inclusion in chemotherapy (days)	61.5 ± 11.4	63.2 ± 13.1	0.73
Concomitant radiotherapy	3 (6.82%)	5 (11.9%)	0.11
Days since first chemotherapy	23.7 ± 12.6	20.4 ± 11.4	0.32
Chemotherapy protocol			0.41
Oxaliplatin + capecitabine	17 (38.64%)	19 (45.24%)	
Fluorouracil + leucovorin	9 (20.45%)	8 (19.05%)	
Oxaliplatin + leucovorin + fluorouracil	7 (15.91%)	9 (21.43%)	
Others	11 (25%)	6 (14.28%)	

Values were expressed as *n* (percentage) or mean ± SD

*BMI* body mass index

The results of QLQ-C30 scales (Table 2) demonstrated no significant improvements with respect to global quality of life (*p* = 0.13), physical function (*p* = 0.084), social function (*p* = 0.47), appetite loss (*p* = 0.52), pain (*p* = 0.61) and diarrhea (*p* = 0.62), at week 12 post-intervention between the self-care education and control groups. However, evident benefits of the self-care education with respect to emotional function (*p* = 0.018), role function (*p* = 0.041) and cognitive function (*p* = 0.038) were observed. Consistent with previous reports, here we also noticed significant decline in role function (*p* = 0.032) and increase in nausea/vomiting (*p* = 0.016), diarrhea (*p* = 0.027) and fatigue (*p* = 0.042) in the control group at week 12 after receiving chemotherapy. Notably, emotional function was remarkably improved in the self-care education group (76.3 vs. 69.2, *p* = 0.026), which highlighted the psychological benefits of the self-care education.

**Table 2:** Patient Reported Outcomes of Quality of Life pre−/post-intervention

Outcomes		Study group		*P* value (t test)
		Self-care education (*n* = 44)	Control (*n* = 42)	
Quality of life—EORTC QLQ30 (scale 0–100)				
Global QoL	Pre-intervention	60.7 (24.1)	62.3 (20.8)	0.52
	Post-intervention	62.4 (19.4)	57.9 (21.4)	0.13
	*p*-value (t test)	0.37	0.46	
Physical function	Pre-intervention	84.6 (18.8)	83.5 (20.4)	0.74
	Post-intervention	82.4 (15.9)	77.6 (19.7)	0.084
	*p*-value (t test)	0.36	0.19	
Emotional function	Pre-intervention	69.2 (24.7)	67.5 (23.9)	0.63
	Post-intervention	76.3 (22.6)	65.2 (23.8)	0.018
	*p*-value (t test)	0.026	0.37	
Role function	Pre-intervention	71.5 (33.7)	70.3 (32.1)	0.52
	Post-intervention	69.4 (28.4)	61.3 (30.2)	0.041
	*p*-value (t test)	0.61	0.032	
Cognitive function	Pre-intervention	79.1 (20.7)	77.4 (18.9)	0.31
	Post-intervention	82.8 (18.7)	73.5 (19.0)	0.038
	*p*-value (t test)	0.56	0.74	
Social function	Pre-intervention	72.5 (25.5)	69.5 (22.7)	0.36
	Post-intervention	70.8 (24.7)	64.6 (21.6)	0.47
	*p*-value (t test)	0.39	0.58	
Nausea and vomiting	Pre-intervention	11.5 (13.6)	13.2 (12.7)	0.34
	Post-intervention	9.2 (16.3)	19.1 (20.3)	0.028
	*p*-value (t test)	0.37	0.016	
Appetite loss	Pre-intervention	8.3 (15.3)	9.2 (16.2)	0.63
	Post-intervention	14.2 (17.1)	16.7 (15.9)	0.52
	*p*-value (t test)	0.39	0.27	
Pain	Pre-intervention	21.6 (31.4)	24.1 (33.6)	0.83
	Post-intervention	25.7 (29.3)	22.6 (37.1)	0.61
	*p*-value (t test)	0.83	0.74	
Diarrhoea	Pre-intervention	13.6 (25.9)	10.3 (22.9)	0.74
	Post-intervention	19.3 (22.7)	18.2 (21.6)	0.62
	*p*-value (t test)	0.032	0.027	
Fatigue	Pre-intervention	17.8 (19.4)	16.9 (23.6)	0.43
	Post-intervention	15.3 (18.6)	21.6 (20.8)	0.029
	*p*-value (t test)	0.48	0.042	

Values were expressed as mean (SD)

QoL:5 Quality of Life;

We further evaluated the fatigue dimensions in detail (Table 3). Although none of the physical, affective and cognitive fatigue was significantly increased, the total fatigue percentage was higher in the control group post-intervention (43.5 vs 38.3, *p* = 0.031), which was significantly improved in the self-care education group (*p* = 0.023). Specifically, the self-care education effectively ameliorated affective fatigue (*p* = 0.011) other than physical and cognitive fatigue. We also analyzed the potential benefits of the self-care education against fatigue with respect to the baseline depression status. We performed depression evaluation at the same time with other indexes. According to the CES-D, there were 19 patients (11 in the self-care education group and 8 in the control group) with baseline depression. Similarly, total fatigue significantly worsened in the control group in the experimental window (38.2 vs. 32.1, *p* = 0.034), which was slightly ameliorated by the self-care education intervention (*p* = 0.027), and this effect was mainly attributed to the benefit on affective fatigue (20.4 vs. 27.3, *p* = 0.016). Notably, the self-care education intervention significantly improved total fatigue in the subgroup of patients who experienced baseline depression (60.2 vs. 66.2, *p* = 0.039), which suggested pronounced effects of this intervention in patients with baseline depression.

**Table 3:** Fatigue pre−/post-intervention

Outcomes		Study group		*P* value (t test)
		Self-care education	Control	
Overall		(n = 44)	(n = 42)	
Total fatigue	Pre-intervention	41.7 (18.4)	38.3 (17.9)	0.76
	Post-intervention	38.7 (20.3)	43.5 (19.2)	0.023
	*p*-value (t test)	0.44	0.031	
Physical fatigue	Pre-intervention	47.1 (25.1)	43.7 (23.6)	0.35
	Post-intervention	47.6 (22.7)	46.1 (24.1)	0.47
	*p*-value (t test)	0.54	0.33	
Affective fatigue	Pre-intervention	33.6 (21.8)	31.4 (19.8)	0.49
	Post-intervention	27.5 (23.7)	33.5 (25.3)	0.027
	*p*-value (t test)	0.011	0.39	
Cognitive fatigue	Pre-intervention	31.3 (19.7)	33.0 (23.1)	0.35
	Post-intervention	29.0 (22.5)	36.2 (20.7)	0.028
	*p*-value (t test)	0.16	0.33	
Without baseline depression		(*n* = 33)	(*n* = 34)	
Total fatigue	Pre-intervention	33.5 (17.1)	32.1 (18.4)	0.63
	Post-intervention	31.5 (19.2)	38.2 (19.7)	0.027
	*p*-value (t test)	0.25	0.034	
Physical fatigue	Pre-intervention	36.4 (21.7)	34.9 (24.1)	0.47
	Post-intervention	38.3 (23.5)	37.4 (23.8)	0.23
	*p*-value (t test)	0.27	0.31	
Affective fatigue	Pre-intervention	27.3 (21.1)	25.8 (20.1)	0.34
	Post-intervention	20.4 (19.4)	27.4 (19.9)	0.019
	*p*-value (t test)	0.016	0.29	
Cognitive fatigue	Pre-intervention	29.2 (18.7)	31.6 (20.5)	0.47
	Post-intervention	26.9 (20.7)	34.9 (21.7)	0.038
	*p*-value (t test)	0.13	0.24	
With baseline depression		(*n* = 11)	(*n* = 8)	
Total fatigue	Pre-intervention	66.2 (15.0)	64.7 (18.3)	0.44
	Post-intervention	60.2 (16.8)	66.2 (16.3)	0.042
	*p*-value (t test)	0.039	0.26	
Physical fatigue	Pre-intervention	79.1 (25.2)	81.3 (22.7)	0.55
	Post-intervention	75.5 (24.3)	83.1 (22.5)	0.13
	*p*-value (t test)	0.29	0.33	
Affective fatigue	Pre-intervention	52.3 (11.6)	55.1 (13.2)	0.44
	Post-intervention	48.9 (13.1)	59.3 (14.0)	0.046
	*p*-value (t test)	0.37	0.28	
Cognitive fatigue	Pre-intervention	37.4 (24.3)	39.2 (21.9)	0.49
	Post-intervention	35.1 (19.5)	41.7 (18.6)	0.041
	*p*-value (t test)	0.12	0.31	

Values were expressed as mean (SD)

## Discussion

Gastrointestinal cancer has increasingly become a chronic disease due to the advances in medical care, which also imposed higher request on the self-care education to improve individual health conditions. According to the Orem’s self-care theory, the nursing knowledge is a foundation for chronic disease care, which greatly depends on the expertise of clinical nurses and varies individually owing to age, economic status, education status, hygiene conditions and intension. In this study, we performed a clinical investigation on the potential beneficial effects of the self-care education in gastrointestinal cancer patients. Our results demonstrated the severe side effects elicited by chemotherapy in the control group of gastrointestinal cancer patients during the intervention window, which included decreased role function, worsened nausea/vomiting, diarrhea and increased fatigue. The self-care education significantly improved the emotional function after 12-week of intervention in comparison with the control group, which highlighted its beneficial effect in mental stress relief in our gastrointestinal cancer subjects. The cancer-related fatigue frequently associated with physical, affective and cognitive factors. We further characterized the significantly worsened total fatigue in the control group despite the lack of remarkable changes in any of physical, affective or cognitive fatigue. The self-care education intervention greatly improved both affective and total fatigue, and the latter effect could be predominantly attributed to the former. We have also taken the depression status into consideration with respect to the fatigue reaction, and our finding highlighted that the self-care education intervention ameliorated total fatigue post-intervention in the subgroup with baseline depression in comparison with the subgroup without baseline depression. Taken together, our experimental investigation demonstrated the beneficial effects of the self-care education in gastrointestinal cancer patients under chemotherapy.

According to Orem’s self-care theory, the self-care education course consists of muscular progressive relaxation, distraction and nutrition optimization [11]. Muscle relaxation was especially effective in improving both pharmacologic and psychological nausea/vomiting complications associated with chemotherapy in cancer patients [14–16]. In agreement with this notion, in this study, our experimental subjects were instructed to relax muscles with a deep breath and finger massage, which showed evident benefits against nausea/vomiting and fatigue during chemotherapy.

Distraction was another well-accepted measure to relieve the severe side effects of chemotherapy [17–19]. Here we composed our self-care education course with music appreciation and meditation to distract the mind in the experimental patients before, during and after receiving chemotherapy. The patients who practiced distraction stayed calm, positive and improved physical function. Our results provided evidences in support of the beneficial effects of distraction against the side effects related to chemotherapy in gastrointestinal cancer patients.

Increasing data have implied that nutrition adjustment is the third way to control the severe side effects of chemotherapy in our self-care education intervention. In our design, patients participating the self-care education were instructed to switch their regular diet into dry, carbohydrate-rich, protein-rich and vitamin-rich foods, which was widely accepted for chronic disease patients as well [20]. Our results unambiguously demonstrated that persistent dietary adjustment significantly improved the quality of life of the experimental subjects in our questionnaire. Noteworthily, our subject patients were asked to keep continuous use of ginger before, during and after receiving chemotherapy. Ginger was widely used in Chinese food as an important spice and was also a multiple facet ingredient in traditional Chinese medicine in the treatment of nausea complications [21, 22]. Consistent with the previous report by Ghanbari et al. [23], our investigation based on the participant’s experience suggested the possible benefits of ginger in control of nausea/vomiting in gastrointestinal cancer patients under chemotherapy.

In summary, our data underlined the necessity of the self-care education for gastrointestinal cancer patients receiving chemotherapy. Notably, the current sample size was relatively limited and a large-scale population is warranted in the following investigations, wherein the confounding factors such as disease stage and therapeutic regimens potentially influencing the analytical results should be strictly controlled. The patients at different stages of the disease might benefit from the self-care education to varying degrees. In addition, certain variables were beyond the scope of this investigation including financial condition, mental and spiritual belief, as well as different interests and motivation levels of the participants.

## Conclusions

Based on our results, the availability of the self-care education was critical in this regard to improve the quality of life in gastrointestinal cancer patients under chemotherapy in all aspects under the questionnaire. Moreover, this intervention appeared necessary for both social meaning and psychological establish as well. Our investigation also highlighted the critical role of nurses in transferring self-care knowledge and providing supports for both patients under chemotherapy and their families [24].

## Supplementary information

**Additional file 1:** The self-care program.

## Data Availability

All data generated or analysed during this study are included in this published article.

## Abbreviations

EORTC: European Organization for Research and Treatment of Cancer
