# Supplementary material for: The effects of add-on self-care education on quality of life and fatigue in gastrointestinal cancer patients undergoing chemotherapy
Source: BMC Complement Med Ther. 2020 Jan 16;20:15. doi: 10.1186/s12906-019-2800-5 (PMC7076856; doi:10.1186/s12906-019-2800-5)
Supplement: Supplementary file 1 — Additional file 1: The self-care program. [file 12906_2019_2800_MOESM1_ESM.docx]

Supplementary Information

The self-care program. The education section composed of 12-session course. At the beginning of each week, one session (45-60 minutes) was carried out. Every session includes three parts:

| muscular progressive relaxation | Finger, arm, leg massage training |
| --- | --- |
| distraction | listen to soothing music or videos |
| nutrition optimization education | frequent meals of small portion, using mashed food without stimulants, using cold food, using rich carbohydrate and low-fat regimen such as roasted bread, cookies, yoghurt, juice, cooked cereals, soft peeled fruits, juicy melon, banana, and other natural fruit juices |
